# Trainee-supervisor collaboration, progress-visualisation, and coaching: a survey on challenges in assessment of ICU trainees

**DOI:** 10.1186/s12909-023-04980-0

**Published:** 2024-02-06

**Authors:** Johannes B. J. Scholte, Johannes C. Strehler, Tatjana Dill, Walther N. K. A. van Mook

**Affiliations:** 1 Department of Intensive Care Medicine, Cantonal Hospital Lucerne, Lucerne, Switzerland; 2 Master of Medical Education Student, University of Bern, Bern, Switzerland; 3 Department of Anaesthesiology and Pain Medicine, Inselspital, Bern University Hospital, Bern, Switzerland; 4 Swiss Air-Ambulance Ltd, Rega, Zurich, Switzerland; 5 Department of Intensive Care Medicine and Academy for Postgraduate Medical Training, Maastricht University Medical Centre, Maastricht, The Netherlands; 6 School of Health Professions Education, Maastricht University, Maastricht, The Netherlands

**Keywords:** Intensive care medicine, Intensive care unit, Assessment, Collaboration discontinuity, Trainee’ progress visualisation, Coaching, Feedback, Dashboards, Shared bedside care, Thematic analysis

## Abstract

**Background:**

Assessing trainees is crucial for development of their competence, yet it remains a challenging endeavour. Identifying contributing and influencing factors affecting this process is imperative for improvement.

**Methods:**

We surveyed residents, fellows, and intensivists working in an intensive care unit (ICU) at a large non-university hospital in Switzerland to investigate the challenges in assessing ICU trainees. Thematic analysis revealed three major themes.

**Results:**

Among 45 physicians, 37(82%) responded. The first theme identified is trainee-intensivist collaboration discontinuity. The limited duration of trainees’ ICU rotations, large team size operating in a discordant three-shift system, and busy and unpredictable day-planning hinder sustained collaboration. Potential solutions include a concise pre-collaboration briefing, shared bedside care, and post-collaboration debriefing involving formative assessment and reflection on collaboration.

The second theme is the lack of trainees’ progress visualisation, which is caused by unsatisfactory familiarisation with the trainees’ development. The lack of an overview of a trainee’s previous achievements, activities, strengths, weaknesses, and goals may result in inappropriate assessments. Participants suggested implementing digital assessment tools, a competence committee, and dashboards to facilitate progress visualisation.

The third theme we identified is insufficient coaching and feedback. Factors like personality traits, hierarchy, and competing interests can impede coaching, while high-quality feedback is essential for correct assessment. Skilled coaches can define short-term goals and may optimise trainee assessment by seeking feedback from multiple supervisors and assisting in both formative and summative assessment.

Based on these three themes and the suggested solutions, we developed the acronym “ICU-STAR” representing a potentially powerful framework to enhance short-term trainee-supervisor collaboration in the workplace and to co-scaffold the principles of adequate assessment.

**Conclusions:**

According to ICU physicians, trainee-supervisor collaboration discontinuity, the lack of visualisation of trainee’s development, and insufficient coaching and feedback skills of supervisors are the major factors hampering trainees’ assessment in the workplace. Based on suggestions by the survey participants, we propose the acronym “ICU-STAR” as a framework including briefing, shared bedside care, and debriefing of the trainee-supervisor collaboration at the workplace as its core components. With the attending intensivists acting as coaches, progress visualisation can be enhanced by actively collecting more data points.

**Trial registration:**

N/A.

**Supplementary Information:**

The online version contains supplementary material available at 10.1186/s12909-023-04980-0.

## Introduction

Intensive care units (ICUs) are unique environments where care is being provided 24/7 to the sickest patients. This results in a high proportion of shift work and large teams of health care professionals, including but not limited to physicians and nurses, leading to frequent changes in team’s composition. It is the responsibility of both the supervising intensivists (assessors) as well as their trainees to optimally facilitate the training process resulting in a successful graduation [1]. In university-level and large ICUs, both rotation residents and fellows are simultaneously present as trainees. This broad span of differences in prior knowledge and clinical experience, comprehension of pathophysiology and special interests makes the design of courses and bedside teaching compliance with and nationally mandated requirements of the speciality training programs significantly more complicated [2]. To motivate the trainees and drive their development, both formative and summative assessment should be used in line with the specific objectives of their learning [3]. Through assessments, the development of each trainee can be monitored and their individual needs and challenges identified and subsequently addressed. Each assessment method bears its own strengths and limitations [4–6]. Since ICU trainees are frequently observed by attending intensivists, the ICU is ideal for workplace-based assessment (WPBA) [6].

In 2003, the Canadian Medical Education Directives for Specialists (CanMEDS) framework introduced seven, now internationally recognised competency-roles for postgraduate medical education [7]. Nowadays in Switzerland, trainees should develop proficiency in all these roles. In 2010, competency based training was introduced in the intensive care medicine training programs by Competency-Based Training in Intensive Care in Europe (CoBaTrICE), a panel of the European Society of Intensive Care Medicine (ESICM), leading to 102 underlying competencies that ICU-fellows should achieve during their training [8]. Furthermore, entrustable professional activities (EPAs), programmatic assessment and milestones all aim to facilitate assessment [1, 5, 9–15]. Whereas these roles, competencies, programmatic assessments, EPAs, and milestones all form robust matrices, transformation of these matrices into an assessment framework that is reliable, valid, and simultaneously feasible in daily practice in the ICU, appears to be extremely challenging [6, 11, 16]. Currently, it is unclear which factors, as perceived by ICU physicians, influence and challenge assessments the most. Consequently, before further transforming these matrices into reliable, valid, and feasible assessment frameworks, it is essential to first identify these perceived challenges.

Hence, we designed a study to explore the challenges and potential solutions regarding ICU trainee assessment, as perceived by ICU physicians. Our results may aid improvement of the assessment frameworks for physicians and educators working in the ICU and additionally could be of interested to other medical specialties with similar challenges in training and teaching, e.g. anaesthesiology and emergency medicine.

## Methodology

### Descriptive qualitative survey

In our ICU, we dedicate many resources to postgraduate education and teaching of our trainees, yet in an annual nationwide anonymous, validated, and published survey regarding the postgraduate training program in our department, we received rather disappointing trainees’ satisfaction rates [17]. After completing a one-week module training in the area of assessment, the primary author had the insight that challenges in assessment might be partly the cause of these disappointing satisfaction rates. Therefore, he therefore constructed a survey consisting of two open-ended questions, allowing ample space for the participants to provide detailed answers:

- “Which challenges in our ICU regarding the assessment of trainees do you see?”
- “Do you have any other concerns?”

The aim was to explore current thoughts regarding perceived and experienced challenges in assessing ICU trainees in the workplace. Therefore, we used a descriptive qualitative research as the main methodology [18]. All trainees and intensivists employed in our ICU were invited to participate. Three groups of participants were included: residents, fellows, or intensivists.

The survey was distributed to the participants in November 2022 with a specific two-week period for completion. After one-week, a friendly reminder was sent to non-responders. During the two-week survey period, some ICU physicians working at the same time as one of the authors (JBS), were verbally made aware of the survey. The primary author (JBS) had full access to all non-anonymised data, which were analysed confidentially and subsequently anonymously encrypted. The other authors analysed the anonymous data whereby the second author (JCS) could, theoretically, have gained access to the non-anonymous data, due to hospital policies.

### Setting and classification of the participants

This survey was performed in a 24-bed ICU, run by 45 ICU physicians (20 intensivists and 25 trainees, corresponding to 34 full time equivalent [FTE]) on average, at the Cantonal Hospital of Lucerne, Switzerland [19]. Supplement 1 shows an organisation chart of all ICU physicians at the time of the survey. The ICU is a teaching centre and serves as a tertiary care centre for a population of approximately 700,000 residents of central Switzerland, maintaining a 24/7 intensivist coverage on-site. All 20 intensivists execute clinical tasks with a 0.5FTE to 0.8FTE employment rate. All physicians working in the ICU are called ICU physicians. A resident is an ICU physician who is not in postgraduate training to become an intensivist and works in the ICU during a rotation of their training as either an internist, an anaesthesiologist, or a surgeon. A fellow, on the other hand, trains to become an intensivist and generally gained extensive experience in anaesthesiology, internal medicine, or both, or has even completed his/her primary medical speciality in one of these disciplines. Both groups of trainees, residents and fellows alike, were assessed. Intensivists are present 24/7 in the ICU and were either attending physicians, leading physicians, senior consultants, or the head of department. The factor common to all intensivists is that they were the clinical supervisors and assessors of the trainees.

### Intensivist’ training in Switzerland

In Switzerland, the intensivist training is six years without the need for the physician to specialise in another medical field. The postgraduate training includes two and a half to four years in intensive care medicine and two to three and a half years in clinical disciplines other than intensive care medicine, with a minimum of 12 months in internal medicine and 12 months in anaesthesiology. Fellows are trained according to the international standard set by CoBaTrICE [8] and the Swiss Society of Intensive Care Medicine [20]. Formative WPBA is performed using Direct Observation of Procedural Skills (DOPSs) and Mini Clinical Evaluation Exercise (MiniCEXs) [6]. Fellows seldomly have a supervisory role over residents. All trainees have a designated intensivist appointed as their tutor.

### Data analysis and author’s contribution

The survey was sent using Microsoft Forms. A response rate of 70% or higher was aimed for [21]. As the survey response rate was high and participants provided rich, detailed and therefore highly granular responses, thematic analysis was suggested as most appropriate framework by the last author (VNM) to best describe the participants’ opinions and perspectives. We followed the framework on thematic analysis founded by Clarke and Braun as elaborated in the Association for Medical Education in Europe-(AMEE) Guide no.131 [22, 23]. Themes were defined as patterned responses or meanings and generated by inductive coding [22]. First, JBS and JCS familiarised themselves repeatedly with the data. Initial codes and suggestions for themes were subsequently created by JBS and his findings were structured using Microsoft Excel. Subsequently, JCS separately applied the same process, adjusting the Excel file. In repeated face-to-face meetings between the first two authors, their findings, ideas, and discrepancies were intensively discussed. To enhance scientific rigour and reduce potential biases, we involved an independent female clinician, TD, who was employed as an ICU trainee in a different hospital at the time of the survey, to independently conduct the thematic analysis. Discussions between these three authors led to the proposition of three themes. Consensus was reached by reviewing the raw data. All the steps were transparently presented to the last author (WNM) who critically analysed these steps and provided constructive inputs for optimisation. After repeatedly revising all steps led by JBS in face-to-face meetings, the themes were defined and named, and then collectively endorsed in a group meeting attended by JBS, JCS and TD. All authors actively participated to the writing process, under the coordination of JBS.

### Reflexivity statement

We appreciate our diverse identifies within the team including gender (one woman and three men), nationality (two Dutch, one South-African, one Swiss), professional roles (one trainee and three intensivists), training environment (two trained in Switzerland and two completed their training in the Netherlands) and specialism (two internist-intensivists, one anaesthesiologist-intensivist, and one anaesthesiologist), among others. We recognise that our individual privileges based upon our different training environments and professional roles influenced our data collection and data interpretation.

The primary author (JBS) is a man, physician, and scholar of medical education with foreign nationality, his demeanour and personal experiences as the initiator of the study, colleague, supervisor, and assessor of trainees, possibly influenced the information shared and withheld by participants of the survey.

JBS, JCS, WNM are experienced intensivists practicing in the ICU. During the study, JBS served as an attending physician, while JCS held a position of leading physician in the hospital. JBS and TD were students following the Master of Medical Education (MME) program at the University of Bern, Switzerland. As previously stated, this study was initiated by JBS out of genuine interest and as part of his post-course assignment for his MME module on assessment and was undertaken as part of a broader need by the ICU physicians regarding WPBA. As the survey was distributed to all ICU physicians in Lucerne, both JBS and JCS completed the survey and are identified as intensivist 1 and 2, respectively. JCS holds an executive Master of Business Administration in Health Care Economics and -Management. He is the educational head of the medical training program of the ICU and, in collaboration with JBS, developed blended learning courses for ICU trainees. TD is a female specialist anaesthesiologist at the University Hospital of Bern, who recently completed her postgraduate training in Intensive Care Medicine at the University Hospital of Bern and is currently employed as a full-time pre-hospital physician involved in the inter-facility transfers of critically ill patients. WNM is a full professor at the School of Health Professions Education and serves as the dean of the Academy for Postgraduate Medical Training. Our common axiological approach is to optimise postgraduate medical training and produce highly competent medical specialists. The ontology and epistemology of this study, which analysed interactions between assessors and trainees, inducting themes, were reflected upon using a constructivists paradigm by JBS, TD and WNM [24]. JCS’s pragmatic approach contributed to the practicality of the study. JBS worked under the guidance of JCS and was a former PhD student of WNM. Our team frequently met in person and online to evaluate incongruent interpretations of the data and to discuss the effects of identity, background, and professional roles on what was shared and withheld by the participants.

## Results

The questionnaire was completed by 37 physicians, resulting in an 82% response rate. Specifically, nine out of 10 (90%) residents, 13 out of 16 (81%) fellows, and 15 out of 19 (79%) intensivists participated into the survey. During the two-week response period, three ICU physicians (one intensivist and two residents) were unavailable. Table 1 provides a demographic description of the participants and the median number of words they used in their responses.

**Table 1:** Participants demographics and number of words per responses

	**Residents**	**Fellows**	**Intensivists**	**Total**
Number of responders (%)	9 (90%)	13 (81%)	15 (79%)	37 (82%)
Age, median years (IQR)	31 (1)	33 (3)	45 (6)	36 (9)
Female, n (%)	3 (33%)	8 (62%)	6 (40%)	17 (46%)
Experience internal medicine, mean months (SD)	30 (17)	35 (20)	31 (26)	NA
Experience in anaesthesiology, mean months (SD)	13 (16)	25 (34)	39 (38)	NA
Experience in surgery, mean months (SD)	0 (0)	2 (5)	3 (10)	NA
Experience in intensive care medicine, mean months (SD)	3 (2)	14 (11)	91 (37)	NA
Question 1, number of words; median months (IQR)	67 (106)	48 (62)	54 (49)	55 (66)
Question 2, number of words; median months (IQR)	0 (21)	55 (72)	4 (15)	10 (34)

*Abbreviations*: *IQR* interquartile range, *NA* not applicable, *SD* standard deviation

The thematic analysis revealed three themes, each consisting of six subthemes. Each theme and subtheme were supported by sufficient inputs from different ICU-physicians, as displayed in Supplement 2. Subsequently, we will elucidate the three themes along with their corresponding subthemes and variations among the three participant groups are explored. In the discussion section, we further elaborate upon the different themes and their implications for the proposed assessment framework.

### Trainee-supervisor collaboration discontinuity

The first theme is “trainee-supervisor collaboration discontinuity”. We defined trainee-supervisor collaboration discontinuity as a situation where the collaboration between trainees and supervisors allows too little observation time for proper assessments. For a specific trainee-intensivist pair, there were mostly only fractions of collaboration-time with much time in between in which collaboration and therefore WPBA was not possible.

**Fig. 1:**
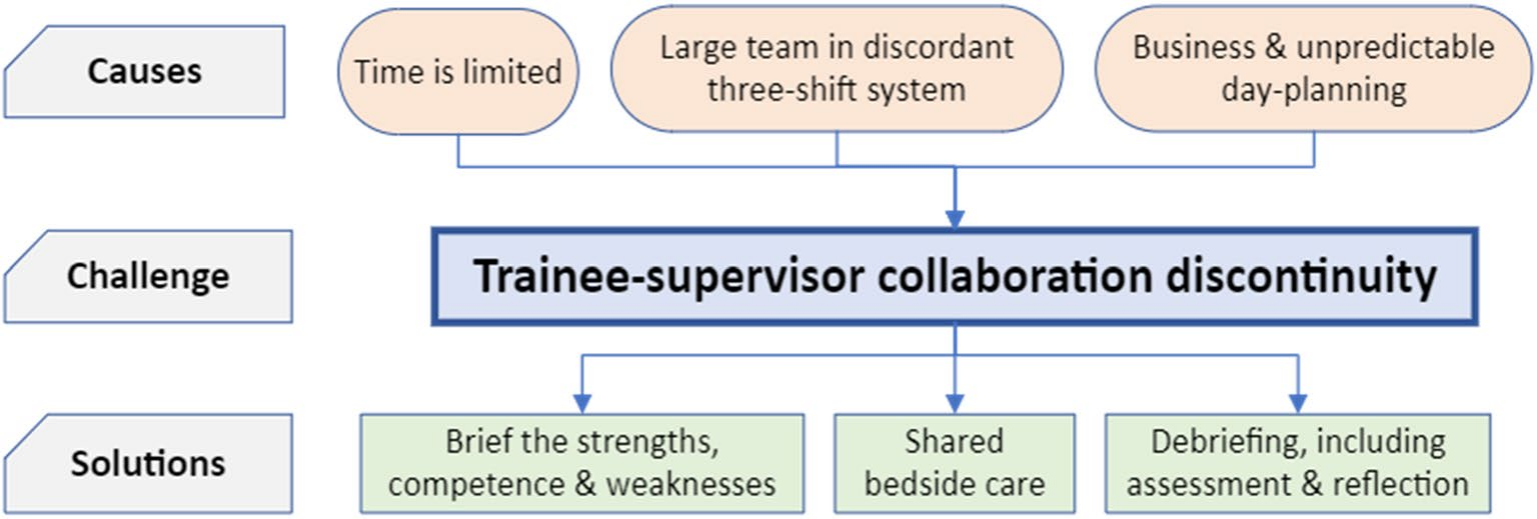
Theme 1: Trainee-supervisor collaboration discontinuity. This figure visualises the first theme; “trainee-supervisor collaboration discontinuity”. Three subthemes contributed to this challenge; limited time, due to short trainee stays, making building trust difficult, a large team working in a discordant three-shift system, and unpredictable day-planning. Proposed solutions included pre-shift briefings to set trainee’s goals, shared bedside care for enhanced observation time, and post-collaboration debriefings that should facilitate workplace-based assessment and reflection. ICU: intensive care unit

We illustrated this theme with six subthemes that delineate the challenges and potential solutions, as depicted in Fig. 1. Three subthemes were identified as potential causes leading to different challenges. Firstly, time is limited: it takes time to get to know and trust each other and this building of trust is challenged by the fact that trainees remain in our ICU for six to 12 months, exceptionally up to 24 months. Secondly, the necessary three-shift system requiring a large team of physicians decreases the chances that a specific trainee-intensivist pair collaborates again soon. Additionally, this shift-duty system of trainees and intensivists was planned independently. Third and lastly, business and notoriously unpredictable day planning lead to asynchrony in bedside work according to our participants. Whereas the trainees are not always available for bedside discussions, intensivists are frequently involved in other activities, such as coordinative and administrative tasks and supervision of multiple trainees simultaneously. Consequently, there seems to be insufficient time for collaborating with trainees to adequately observe progression, strengths, and deficits of the individual trainees. Participants offered potential solutions, which are depicted in three subthemes in Fig. 1. Firstly, a short briefing before collaboration to inform the supervisor about strengths and weaknesses of the trainee can provide insight into the trainee’s competencies, goals, and needs. Secondly, some activities take place independently without direct observation by either the intensivist or the trainee. When trainees and intensivists share the bedside care, observation time increases, therefore facilitating formative assessment. Finally, debriefing after a longer or more efficient collaboration period between trainees and intensivists should induce workplace-based assessment by MiniCEX, DOPS, EPA, and/or (narrative) feedback, and reflection upon the collaboration.

### Trainees’ progress visualisation

**Fig. 2:**
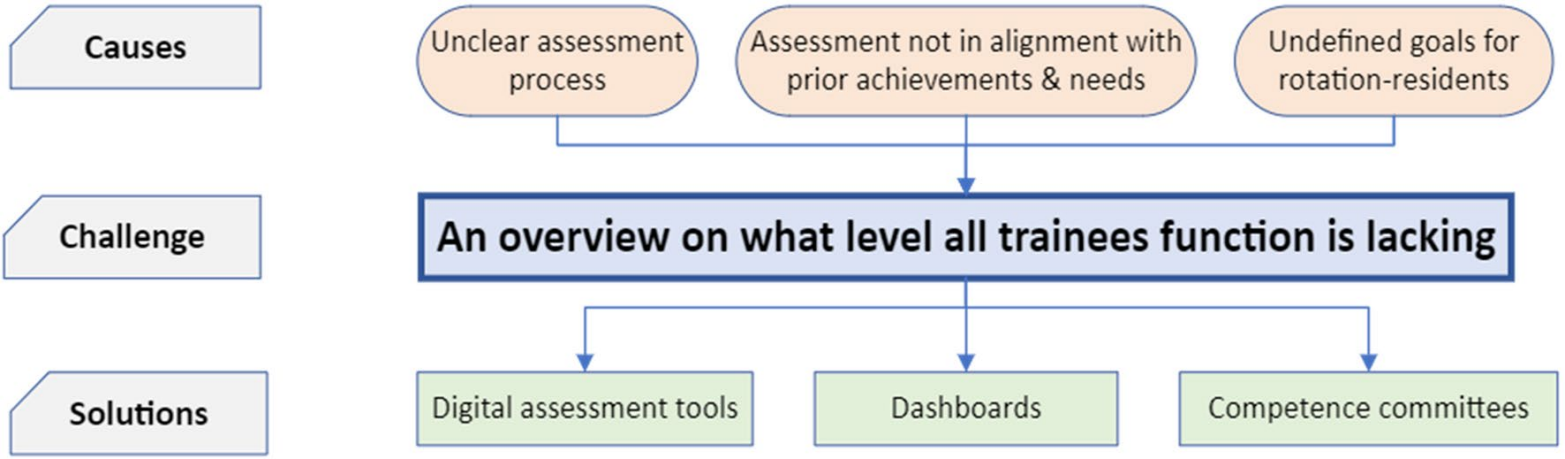
Theme 2: Progress visualisation. In this figure, the second major theme, progress visualisation, is presented. Three subthemes pose challenges: unclear assessment criteria, assessment not in alignment with previous observations, and unclear expectations for rotation-residents from other medical specialities regarding achievements. Three subthemes provide potential solutions; digital assessment tools, dashboards, and competence committees

The second theme is “trainees’ progress visualisation”, which is defined as the wish for a comprehensive dashboard that includes representations of trainees’ competence levels, encompassing their strengths, weaknesses, and goals. This theme is graphically displayed by Fig. 2. Three subthemes were identified that potentially lead to a lack of overview on what level trainees thrive and therefore contribute to this challenge. First, the methods used for assessment are apparently unclear to the supervisors. Second, the assessments were not always in alignment with prior observations and level of competence, prior achievements, specific needs, and pre-defined goals. Finally, in contrast to the fellows, there is a lack of clarity regarding the competency goals and expectations for rotation residents from various other medical specialities.

Three subthemes provide potential solutions, as suggested by our participants. First, there is a desire for the implementation of a digital assessment tool and an electronic portfolio (e-portfolio). An assessment tool can make assessment easy and pleasant. The input of the e-portfolio should be generated by the digital assessment tool to monitor progression, strengths, weaknesses, and goals. Second, fed by data points collected by the digital assessment tools, dashboards may offer a comprehensive visualisation of the trainees’ progression. Lastly, intensivists might conduct meetings in which the trainees’ progression is being discussed and evaluated, known as competence committees. This meeting would have a solid base to conduct the summative assessment using a dashboard created by an electronic portfolio to visualise data points regarding learning progression, level of competence, strengths, weaknesses, goals, and recommendations. Resident 7 and intensivist 14 suggested to use level of trust in the assessment (see theme 2 subtheme 4 in supplement 2).

### Insufficient coaching & feedback

The third theme is “insufficient coaching and feedback” and is defined as both unsatisfactory coaching and unsatisfactory feedback resulting in an inadequate assessment of the trainee. Although a designated tutor was perceived to be a potentially powerful way to improve the quality of assessment, implementation of the tutor system did not meet the expectations of ICU fellows and intensivists, primarily due to collaboration discontinuity, as suggested by the first theme.

**Fig. 3:**
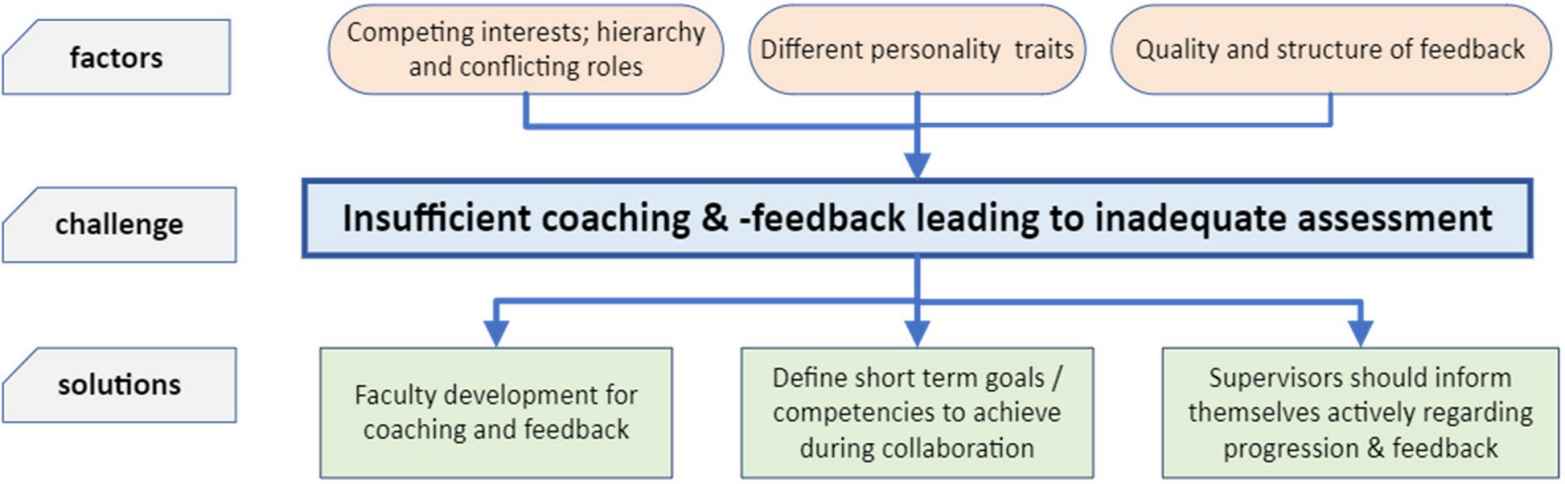
Theme 3: insufficient coaching & feedback. In this figure, the third theme is visualised. Three factors may contribute to insufficient coaching and feedback. These factors include hierarchy and conflicting roles (competing interests), personality traits and the quality and structure of feedback. According to our participants, potential solutions consist of faculty development programs, short term goal setting for clinical pairs, and ensuring supervisors’ knowledge on the progress of their trainee’s development

The theme of insufficient coaching and feedback is illustrated, along with its six subthemes, in Fig. 3. Trainees seek feedback that is constructive, frequent, provided by all supervisors, selected nurses, and to be facilitated both by the trainee as well as by the supervisor. Three factors influencing (in)sufficient coaching and feedback were identified. Firstly, hierarchy (determining who takes precedence in performing invasive procedures) and conflicting roles (balancing the roles of a clinician and a teacher) may lead to dissatisfaction in coaching. Regarding invasive procedures, potential WPBA moments are lost when the intensivist decides to perform the procedure themself. Second, personality traits between trainee and intensivist do not always align and are not always facilitating optimal supervision and coaching. Third and last, effectiveness of the feedback was perceived to depend on its quality, suitability, and the structure in which it is provided. Three potential solutions were given by the participants. First, intensivists demand faculty development and education in coaching and giving feedback. Second, short term goals and / or achievable competencies should be defined before short periods of trainee-intensivist collaboration to induce a form of WPBA. Third and last, designated coaches / tutors should actively inform themselves regarding their trainee’s development and contribute to summative assessment.

### Differences between groups

Supplement 2 also displays the differences between the input of residents, fellows, and intensivists. All three themes were supported by all ICU physicians. The desire for briefing and debriefing, bedside teaching and learning conversations came solely from the trainees, whereas the idea of competence committees and the wish for faculty development regarding teaching came from intensivists alone.

## Discussion

To the best of our knowledge this is the first qualitative study among ICU physicians exploring the challenges in assessing ICU trainees. It revealed that discontinuity in trainees’ collaboration with supervisors, progress visualisation, and the importance of coaching and feedback are the three major themes regarding ICU trainee assessment. These themes not only explain the challenges of assessment but also provide insights into potential solutions.

In the following sections, we delve into the three major themes, present hypotheses on the results, contextualise them with the existing literature, offer additional recommendations for ICU assessment, including a concise collaboration assessment framework, discuss the strengths and limitations of this study, followed by a conclusion.

### Trainee-intensivist collaboration discontinuity

The first theme, prominently and extensively reported by all ICU physicians, highlights the lack of continuity in the trainee-intensivist collaboration. This deficiency is primarily attributed to the subthemes elucidated in the first three blocks in Fig. 1. This theme underscores the deficiency in establishing a robust and enduring longitudinal relationship between trainees and intensivists. As recently demonstrated by Barnhoorn et al., building trust and bonding between trainees and supervisors requires time and investment [25]. However, due to conflicting schedules, the opportunity for trainees and supervisors to work together for extended periods, akin to a trainee-intensivist partnership, is hardly possible. In other countries, the duration of ICU trainee rotations may be as short as one month, emphasising the relevance of our first major theme, trainee-supervisor collaboration discontinuity, in the international context. In contrast to the ICU, trainees and supervisors in other departments ward tend to work together for many months, fostering a more intense longitudinal and continuous relationship which increases the opportunities for learning. Twenty years ago, ICU physicians used to work up to 80 h per week [26], providing collaboration continuity between (the few) trainees and their (few) supervisors. To account for a growing patient population in need of specialist intensive care medicine and more invasive treatments regimes as well as improve work-life balance, physicians nowadays commonly work part-time in larger ICU teams [27], reducing the frequency and duration of collaboration between individual trainees and intensivists. Working in the ICU for a short period under conditions of trainee-intensivist collaboration discontinuity may lead to unclear expectations regarding trainees’ goals and level of supervision needed, unnecessary worries, and frustration among trainees and supervisors [28]. Indeed, less frequent, and shorter trainee-intensivist collaboration might consequently yield less valid and reliable formative assessments, as discussed further in theme two.

Systematic pairing of trainees with intensivists was suggested as a potential solution by some participants, but this was considered unfeasible by our intensivists due to scheduling difficulties and requests, childcare responsibilities and regulations, and other non-clinical activities. Other potentially effective solutions and recommendations regarding the challenge of trainee-supervisor collaboration discontinuity include briefing, shared bedside care, and debriefing. By initiating a short meeting before the trainee-intensivist collaboration starts, the appropriate WPBA and the goals aimed for can be defined and it is more likely that WPBA and the goals will be achieved [6, 29, 30]. Shared bedside care may be defined as a trainee and a supervisor acting in coordination: the discussion of patient cases and therapeutic decisions taking place with both the trainee and the intensivist present. This may lead to more bedside teaching time, enhancing learning and assessment by perceiving more information relevant to performance [31], which is very much appreciated by trainees according to this survey.

At the end of a (short) collaboration, a debriefing may take place as learning conversations that could lead to a formative WPBA and conclude with a moment of reflection upon learning and teaching together [6, 32–34]. This can be approached according to the “debriefing with good judgement" called for by Rudolph et al [35].

### Progress visualisation

For the second theme on the lack of the overview of progress, it is paradoxical that ICU physicians receive daily handovers with graphic representations of trends in haemodynamic, respiratory, neurological, and infection data from all critically ill patients, but they do not receive similar overviews of their trainees’ standing, progressions, and needs [16].

As discussed in the first theme, the validity of assessment may be diminished due to collaboration discontinuity. This reduced validity of assessment may lead to the development of lack of competence for certain trainees [36]. A phenomenon known as “case specificity” can occur, where a trainee performs well in one case but struggles in another [36, 37]. These trainees may lack certain competencies going unnoticed, posing risks to patients, the ICU team, and the trainees themselves. Furthermore, inappropriately supervised trainee activities of a trainee may derive from trainee-intensivist collaboration discontinuity that leads to unfamiliarity with the level of competence of a relatively unknown trainee, as discussed in the first theme*.* Inappropriate level of supervision may have devastating consequences with respect to patient safety and should be avoided at any time.

Therefore, prior assessments should be accessible to other assessors and sampling across assessors should be initiated to enhance reliability of the assessment [37]. Indeed, an easy-to-understand graphic overview is highly desired for optimal assessment by our participants. Currently, data analytics management systems are being developed visualising the trainee’s progress in a comprehensive dashboard [38]. These dashboards can assist competence committees with their summative assessment of an individual trainee [39]. When assessors lack familiarity with a trainee’s performance level, the support, feedback, and subsequent assessment provided, can consequently be insufficient and inappropriate. Before attending the ICU, trainees have worked in other fields where they were similarly assessed [40]. Dashboards that graphically display previous assessments, including strengths, weaknesses, and goals of the newly introduced trainee might be helpful and can be used as a good starting point for planning of further development and appropriate assessment [38, 39, 41]. These dashboards may also display various achieved milestones and level of trust as expressed per different EPAs [1, 9, 12–15, 42] and highlight gaps, or development opportunities, in the trainee’s development. To nourish the dashboards with sufficient data points, frequent short assessments, the use of EPAs [12] and a transparent, easy-to-use, and valid assessment tool may be valuable [13].

Besides documentation of the formative assessments, a dashboard should be able to display achievements in ultrasound courses, simulation education, and the attendance of proposed theoretical courses [43–46].

Frequent changes to teams with diverse backgrounds may lead to unclear expectations for trainees and intensivists [28]. Trainees may feel frustrated proving themselves to “new” assessors repeatedly. Providing evidence of competence can lead to an adjusted level of supervision and trust. Therefore, an effective assessment tool is crucial, the competencies which rotation-residents should acquire during their ICU rotation should be defined, along with the assessment criteria used [47].

### Insufficient coaching and feedback

According to our ICU physicians, the designated tutor should be well-informed about their trainee(s) and actively seek feedback to gain a comprehensive overview of their progress. In postgraduate medical education, supervisors can assume roles as mentors, tutors, and/or coaches. Mentoring involves a directional and longitudinal relationship, induced by the trainee [48, 49]. A longitudinal mentorship program might be beneficial for trainees in different fields in healthcare [50–53]. Peer-mentoring, a specific form of mentoring, might provide multiple benefits when trainees are paired with equally experienced trainees with similar personality traits [54]. Although the best model is yet unknown [55, 56], the ESICM NEXT mentorship program provides practical steps, a checklist, and further guidance for creating and introducing mentorship programs in the ICU [51]. While tutors have specific (clinical, technical) teaching assignments, coaches adopt a more multidirectional approach, identifying strengths and weaknesses and providing insights and guidance [57–59]. In fact, our trainees prefer intensivists to act as coaches rather than traditional tutors.

The notorious sentence *“you did well, just gain more experience”* is unwanted, even loathed, feedback, since trainees learn best from tangible feedback that is allocated to a specific situation [60]. When coaches are trained to provide feedback, learning will be enhanced [61, 62]. Furthermore, coaches are more likely to correctly use the superior construct-aligned scale, as used in Mini-CEX or in EPAs [37, 63]. Revealing an ICU coaching framework to enhance feedback, teach the teacher sessions as part of faculty development programs, a proactive attitude enabling the trainee to achieve their goals, and an active involvement in the summative assessment of the trainee should all be considered [48].

As future intensivists will be part of a large team of healthcare workers, it might be helpful to incorporate the perspectives of other professionals, such as nurses, physiotherapists, logistic employees, even the cleaning staff, patients, and their family members in form of a 360-degree feedback to collect data points that can be visualised in the e-portfolio and dashboard [64].

### Differences between groups

Both residents and fellows appreciate bedside teaching and learning conversations. By initiating shared bedside care, an intensivist can better assess certain competences of the trainee [65], e.g., the level “knows” and “knows how” of the famous Miller’ pyramid [66].

### ICU-STAR framework

**Fig. 4:**
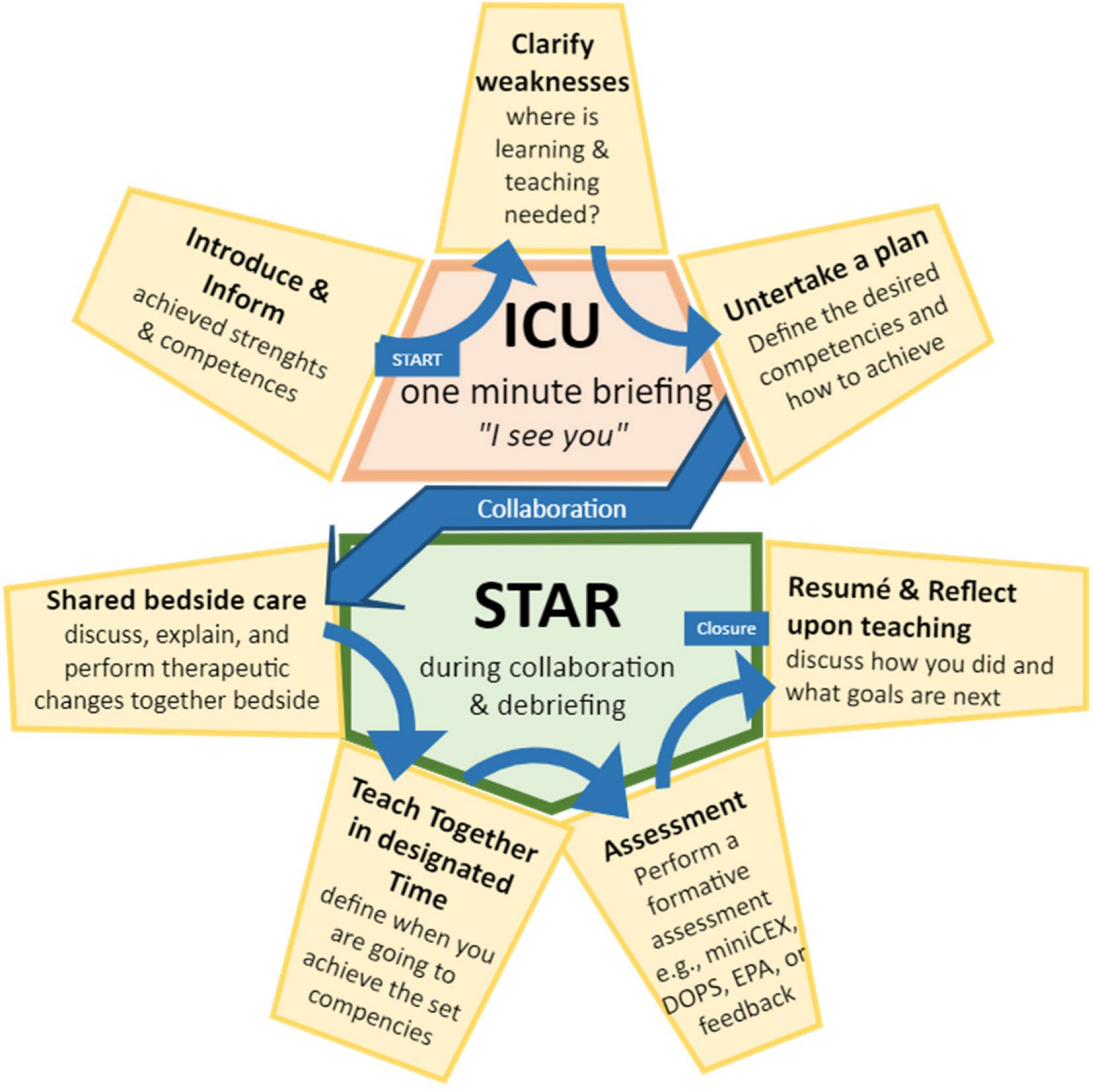
The ICU-STAR framework. This figure presents a potentially effective and robust assessment framework for non-longitudinal short collaborations between trainees and intensivists, which are often the case in the ICU due to factors discussed in theme 1 and 2. The framework commences with “ICU”, which can also be pronounced as “I see you,” signifying the acknowledgement of the trainee by the supervisor and the recognition of collaboration possibilities. The trainee introduces their strengths and weaknesses, and together, the trainee-intensivist duo creates a plan for achieving the targeted competencies during their forthcoming days of collaboration. This process, the “ICU”, can be completed within one or two minutes and can be seen as an opening conversation (briefing) before collaboration. During collaboration, therapy changes and (interprofessional) discussions should ideally occur involving both the trainee and supervisor simultaneously at the bedside (shared bedside care). Time should be designated for teaching, e.g., through bedside education and discussions, to attain the desired competencies. During the debriefing, a formative assessment modality can be completed, and reflection on collaboration, learning, and teaching should conclude the collaboration. Overall, the ICU-STAR framework aims to create awareness regarding the relevance of assessment, offers the trainee a degree of control, and initiates more contact moments between trainees and intensivists leading to a higher-quality assessment

Reflecting on the recommendations and potential solutions derived from all three themes, a discussion meeting was conducted at our hospital, led by JBS, with the participation of 11 ICU trainees and our head of department. Inspired by their additional input and the validated emergency medicine teaching tool “ED-STAT” by Sherbino et al. [67], we have developed “ICU-STAR” as a framework to optimise assessment during relatively short trainee-intensivist collaborations (see Table 2 and Fig. 4). It might be regarded as a short-term solution to simultaneously deal with all three challenges. The “ICU” may be pronounced as “I see you” and should be accomplished during the briefing to set and align expectations, progress, and goals. The “STAR” is executed during shared bedside care, teaching, and debriefing. It includes formative assessment and reflection.

**Table 2:** ICU-STAR

			Theme
**Briefing**			
I	**I**ntroduce &** I**nform	Tell the supervisor about your achieved competencies & strengths	1Sub4, 2Sub2
C	**C**lassify weaknesses	Identify where learning potential is available	1Sub4, 2Sub2
U	**U**ndertake a plan	Define what competencies you want to develop during your collaboration	1Sub4, 2Sub2
**During collaboration and debriefing**			
S	**S**hared bedside care	Discus, explain, and perform therapeutic changes together at the bedside	1Sub5
T	**T**each **T**ogether in the right **T**ime	Determine when you’re going to achieve the defined targets / competencies	1Sub5
A	**A**ssessment	Perform a formative assessment, e.g., MiniCEX, DOPS, EPAs or feedback	1Sub6, 2Sub4, 3Sub5
R	**R**esumé & **R**eflect upon teaching	At the end of the collaboration, discus how you did and what goals will be next	1Sub6, Theme 3

“ICU-STAR” is an acronym to use when trainee-intensivist collaboration is rather short and might be an effective way to ensure assessment. The trainee and the intensivist should both initiate and try to accomplish the “ICU-STAR”. “ICU-STAR” is derived from all major themes (see Supplement 2) and inspired by “ED-STAT”, a validated teaching tool for emergency medicine [67]

*Abbreviations*: *DOPS* Direct Observation of Procedural Skills, *EPA* Entrustable Professional Activity, *Mini-CEX* Mini Clinical Evaluation Exercise

### Strengths, limitations, and further recommendations

With a response rate of 82%, the survey achieved high participation. Participating residents, fellows, and intensivists provided very rich data with consistent challenges across all themes. All applicable consolidated criteria for reporting qualitative research were verified and met [68]. Addressing the issue of collaboration discontinuity is crucial in similar ICUs and may be equally important for other acute care specialists, such as emergency physicians and anaesthesiologists, although not directly acknowledged by their residents [69].

This study has several limitations. First, the survey sample consisted of 37 participants from a single non-validated survey conducted in a single ICU. Therefore, our findings and recommendations should be externally validated in larger and more clarifying studies before general implementation. Second, the first two authors were in a position of power towards the trainees and the responses were non-anonymous, which could have influenced the honesty of the responses. Conducting semi-structured interviews by an independent investigator until saturation has been reached might have been a more optimal method but was not feasible in this context. Third, we specifically sought for challenges, not solutions. Whereas many participants contributed valuable potential solutions, which are included into our manuscript, it is possible that some participants have had brilliant solutions but chose not to share them because we did not explicitly ask for them. Fourth, one might argue that content analysis would have been sufficient and easier [70]. However, based on the detailed and constructive responses, our “open-minded” constructivist paradigm led us to inductive thematic analysis, as we had the freedom to individually validate and weigh the given quotes. Fifth and last, the ICU-STAR framework is not yet validated and does not address all challenges emerging from this study.

We believe it is important to regularly inquire with physicians about their perceived challenges. Indeed, after this survey, assessment became a more discussed topic in our ICU, and discussing assessment may trigger a desired culture change. Following the survey, “ICU-STAR” gained prominence in our ICU, receiving positive evaluations from both trainees and supervisors. Future studies on the ICU-STAR framework should prioritise on (external) validation and the exploration of its contributing factors that facilitate assessment. Besides the practical recommendations derived from this study, we suggest that future researchers in the field of ICU trainee assessment consider trainee-intensivist collaboration discontinuity when evaluating assessment, feedback, assessment tools, or EPAs. Finally, we recommend that societies of intensive care medicine valuate and recommend superior assessment tools and dashboards, further develop these applications, and make them freely available to all ICUs with a dedicated postgraduate training program to facilitate a more uniform assessment of trainees.

In conclusion, trainee-supervisor collaboration discontinuity, progress visualisation, and coaching and feedback were defined as the major factors hampering optimal assessment of ICU trainees by their supervisors. These factors provide direction to further scaffold the assessment framework for ICU physicians, among others by use of “ICU-STAR” framework for short collaborations, digital assessment tools, dashboards, and investing in trained coaches for the trainees.

### Supplementary Information

**Additional file 1:** **Supplement 1.** Organisational chart of our ICU including the number of persons and full time equivalent (FTE) at the time of conduction of the survey. Chief, leading physicians and attending physicians all are intensivists. Fellows and residents are the trainees. **Supplement 2. **Quotes supporting the three themes and their subthemes.

## Data Availability

The raw dataset generated during the current study (being de-identified) is available from the corresponding author on reasonable request.

## Abbreviations

AMEE: Association for Medical Education in Europe
CanMEDS: Canadian Medical Education Directives for Specialists
CoBaTrICE: Competency-Based Training in Intensive Care in Europe
DOPS: Direct Observation of Procedural Skills
EPA: Entrustable Professional Activity
ESICM: European Society of Intensive Care Medicine
ICU: Intensive care unit
ICU-STAR: Introduce/Inform, Clarify, Undertake, Shared bedside care, Teaching Together in Time, Assessment, Resumé / Reflect.
IQR: Interquartile range
Mini-CEX: Mini Clinical Evaluation Exercise
MME: Master of Medical Education
SD: Standard deviation
WPBA: Workplace-based assessment
