# Supplementary material for: Trainee-supervisor collaboration, progress-visualisation, and coaching: a survey on challenges in assessment of ICU trainees
Source: BMC Med Educ. 2024 Feb 6;24:120. doi: 10.1186/s12909-023-04980-0 (PMC10848472; doi:10.1186/s12909-023-04980-0)
Supplement: Supplementary file 1 — Additional file 1: Supplement 1. Organisational chart of our ICU including the number of persons and full time equivalent (FTE) at the time of conduction of the survey. Chief, leading physicians and attending physicians all are intensivists. Fellows and residents are the trainees. Supplement 2. Quotes supporting the three themes and their subthemes. [file 12909_2023_4980_MOESM1_ESM.docx]

**Supplement 1**


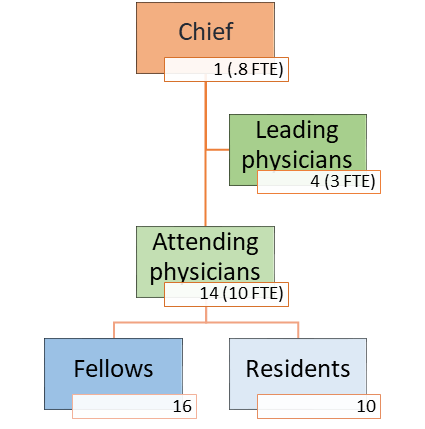


**Supplement 1.** Organisational chart of our ICU including the number of persons and full time equivalent (FTE) at the time of conduction of the survey. Chief, leading physicians and attending physicians all are intensivists. Fellows and residents are the trainees.

**Supplement 2. Quotes supporting the three themes and their subthemes**

| **Theme / Subtheme** | | | | **Who stated this** | **Quotes** |
| --- | --- | --- | --- | --- | --- |
| **Major theme No.1: Trainee-intensivist collaboration discontinuity** | | | | | |
| Definition: A situation where the collaboration between trainees and supervisors allows too little observation time for an proper assessments. | | | | | |
|  | R4 | | “Assessment not possible in the absence of continuous care [from intensivist] and often absence [of the intensivist] for ward rounds or invasive procedures” | | |
|  | F1 | | “There are many trainees and intensivists, three shifts’ schedules, and part-time working intensivists. Consequently, an intensivist rarely sees the individual trainees, sometimes working together less than once a month, which makes it difficult to gain a comprehensive perception of the skills, knowledge and working methods of the respective trainee, especially when there is still a lot to do and sometimes you don't even really get to discuss the patients together.” | | |
|  | F2 | | “Organisationally (shift changes), there is little consistency in the team of individual physicians, as compared to the normal ward, where collaboration lasts for weeks.” | | |
|  | F11 | | “Often only snapshots, intensivists are not present during rounds and frequent change” | | |
|  | I10 | | “One should evaluate residents with whom one had hardly, or not at all, worked. This is due on the one hand to working on a reduced FTE, but mostly to the scheduling. According to the random principle, it works or not that you are assigned together.” | | |
|  | | | **1Sub1: Time is limited; it takes time to know and trust each other and residents stay only six months in our ICU** | | |
|  | R4 | | “Due to the mostly short rotations and the necessary “settling-in time”, residents do not have the idea or the courage to ask for feedback, especially at the beginning”. | | |
|  | R6 | | “Very many combinations of trainees and intensivists, needs a lot of time to get to know each other and build trust.” | | |
|  | R8 | | “I am only there for six months. The rotation to the ICU is like coming fresh from the state exams. I could imagine that the learning curve rises steeply at the beginning and then flattens out. By the time the curve starts to rise again, you're no longer on the ICU as a rotation assistant.” | | |
|  | F6 | | “Getting to know each other is necessary -> otherwise you only have “snapshots” in assessment” | | |
|  | I1* | | “The resident is only shortly here.” | | |
|  | | | **1Sub2: We work in a large team in discordant three shift system** | | |
|  | R1 | | “Frequently changing trainee-intensivist combinations makes comprehensive feedback difficult”. | | |
|  | R2 | | “We are working in shift schedules with a large team of intensivists” | | |
|  | R6 | | “The biggest challenge, in my opinion, is the large team with constantly changing supervision of the trainees.” | | |
|  | F2 | | “Presumably, it is not easy to obtain a homogeneous picture of the skills, competences, deficits and progress of the trainees, given the large numbers of trainees, and, in individual cases, usually little common working time.” | | |
|  | F4 | | “Due to frequent changes in the collaboration of different trainees with different intensivists (due to shift schedule), it seems difficult that each intensivist knows the current status of the trainee in terms of knowledge and technical skills and can challenge /help him/her accordingly” | | |
|  | F12 | | “Rapidly changing teams.” | | |
|  | F13 | | “To be able to assess trainees, you need time and commitment. You must spend time with them and look after them. Only in this way can you recognise their strengths and weaknesses; promote the former and support them in improving the latter. Ideally, professional and personal development takes place and can be evaluated.” | | |
|  | I2* | | “There is little consistency in scheduling. We can only observe trainees for a short time.” | | |
|  | I5 | | “Observations made during one shift only.” | | |
|  | I6 | | “Few shift duties together with certain trainees, all aspect cannot be assessed.” | | |
|  | I4 | | Sometimes, we don’t work together long enough to be able to assess manual skills or you haven’t “experienced” resuscitation or the care of very critically ill patients together”. | | |
|  | I8 | | “We work in shifts and in a large team, so it is rare to work continuously with a trainee for several days to get a good picture” | | |
|  | I9 | | “Due to the shift-duty system, we typically work with a few trainees frequently and with others almost not at all.” | | |
|  | I11 | | “Large team with often only single shifts with the specific trainee.” | | |
|  | I13 | | “Big team, I can’t judge some trainees because we worked together too little” | | |
|  | I14 | | “Very different shift-overlaps. Often the impression of the last shift remains decisive, but an assessment over the entire period is difficult, also because of the different shift overlaps.” | | |
|  | I15 | | “With the trainees I work with more often, the assessment is not a problem. With some trainees I have only worked on individual shift at longer intervals - here I find assessment very demanding.” | | |
|  | | | **1Sub3: Busyness and unpredictable day planning** | | |
|  | R1 | | “With a sometimes-heavy workload for the intensivist, time for feedback is limited” | | |
|  | R3 | | “Sometimes there is not enough time for more detailed assessments, discussions, or teachings, especially in the late shift, when the intensivist has to look after two ICUs at the same time and there is a lot going on.” | | |
|  | R5 | | “I think this (=assessment) is very individual, and also includes factors that we cannot influence (e.g., when there are many patients, a lot going on, many transfers, etc.). | | |
|  | F7 | | “We need bedside-teaching, yet intensivists are frequently very busy” | | |
|  | I1* | | “It is always busy in the ICU” | | |
|  | I7 | | “In the role of medical manager, one is strongly occupied by phone calls, clarifications and consults on the ward.” | | |
|  | I14 | | “The is often time-pressure in duties so we cannot wait for own decisions/inputs from trainees, which makes assessment difficult later on.” | | |
|  | | | **1Sub4: Briefing before shift-duty: to merge ideas, goals, and expectations as “trainee-handover”** | | |
|  | R5 | | “I think above all that good communication is the most important and most difficult challenge.” | | |
|  | F12 | | “Brief discussion at the beginning of the shift. Are trainees ready for their tasks and aware of what is coming up during the day?” | | |
|  | I2* | | “As an intensivist, discussing with the trainee their plans/suggestions for the patients at the beginning of the shift and intermittently during potentially challenging tasks, can make it easier to assess their working style and experience.” | | |
|  | | | **1Sub5: shared bedside care** | | |
|  | | F6 | “Many of the (patients)interactions (visits, interventions, communication with relatives or consulting disciplines) take place independently. Feedback must therefore be sought actively.” | | |
|  | | F12 | “When intensivists go to see the patients, this should be used as an opportunity to involve the trainee. Not “I prescribed…” as announcement” | | |
|  | | | **1Sub6: debriefing: A longer or more efficient trainee-intensivist collaboration should induce WPBA, feedback and reflection** | | |
|  | F4 | | “Where long-term assessment and detailed feedback are difficult due to the system, it would be even more helpful to establish a short (two minutes) feedback including constructive criticism at the end of each shift or after specific situations” | | |
|  | F12 | | “No “debriefing” after the end of the shift, hardly any feedback on reports / progress-notes.” | | |
|  | F13 | | “A longer block with the same combination of trainee-intensivist could be an opportunity for a short feedback session.” | | |
| **Theme No.2: Progress visualisation** | | | | | |
| Definition: an overview on what level all trainees function is lacking | | | | | |
|  | | F4 | “Due to frequent changes in the collaboration of different trainees with different intensivists (due to shift schedule), it seems difficult that each intensivist knows the current status of the trainee in terms of knowledge and technical skills and can challenge /help him/her accordingly” | | |
|  | | I9 | “With the relatively large number of residents and fellows, it is difficult to keep track of their respective training levels and competencies.” | | |
|  | | | **2Sub1: Unclear assessment process** | | |
|  | F9 | | “Only one appraisal-conversation (after 1 month at the ICU (!)), after that no more official conversations were planned or offered, despite requests. It is unclear what the final [summative]evaluation ultimately refers to.” | | |
|  | I2* | | “We received hardly any training on how to assess trainees” | | |
|  | I5 | | “Inconsistent [assessment] criteria are used” | | |
|  | I6 | | “Assessment is subjective, clear objective points are difficult to define” | | |
|  | | | **2Sub2: Assessment is not always in alignment with previous observations, - level of competence, specific needs, and pre-defined goals** | | |
|  | R1 | | “Very different previous experiences of residents make comprehensive feedback difficult”. | | |
|  | R7 | | “I find self-assessment difficult as a less experienced resident on the ICU, because I don’t understand what I don’t know, especially at the beginning (“Mount stupid”). I therefore think for other to assess me must be even more difficult.” | | |
|  | R9 | | “Know the individual training status and based on this status, give appropriate and useful feedback.” | | |
|  | F2 | | “Some intensivists would like more control; others would like more independence”. | | |
|  | F3 | | “There are no specific questions about diagnostic considerations and therapeutic plans (“how would you do it?”). I got these questions once in 11 months from a junior intensivist.” | | |
|  | F4 | | “Checking the level of knowledge may not be wrong and may provide the intensivist a lot of information about this level. I would be grateful if knowledge was checked in the sense of teel me what you know what your clinical idea is, and what your idea of a goal-oriented therapy is” | | |
|  | F5 | | “We need feedback according to level of training in everyday life” | | |
|  | F7 | | “The knowledge of the intensivist regarding the level training of the trainee is oft missing” | | |
|  | F12 | | “Respond to the needs of trainees. Some stalk forward carelessly and full of energy. Others need more protection and support – this may also be asked for if not clearly felt.” | | |
|  | F13 | | “To be able to assess trainees, you need time and commitment. You must spend time with them and look after them. Only in this way can you recognise their strengths and weaknesses; promote the former and support them in improving the latter. Ideally, professional and personal development takes place and can be evaluated.” | | |
|  | F13 | | “Detailed determinations at the start of the ICU period with clear learning goals given over a certain period. A motivation or at least structure could provide controllable "successes". | | |
|  | I14 | | “Sometimes there are no clear individual goals” | | |
|  | I15 | | “Perhaps in some cases (e.g., fellows), an oral exchange among the intensivists about the performance would also help. In discussions, I can formulate my impression even better than in written form – although this kind of discussion is very time-consuming.” | | |
|  | | | **2Sub3: Expectations what rotation-residents of different specialities should accomplish during their time in the ICU are not clear** | | |
|  | R5 | | “Individually, I mean that the challenge is that we all have very different levels of training (for example, I still know almost nothing about intensive care medicine). In my opinion, people like me are dependent on active support from the senior physicians in the initial phase, so that we can swim in the right direction, so to speak. In a second step, this would make the assessment easier (after a certain interval), as we can then get a bit of routine and acquire (or try to acquire) the most important features in intensive care medicine. In the beginning, a lot of things are completely new, and it takes time (I’m talking about me) until one really has a plan for oneself and can practise/exercise certain activities in a relaxed and concentrated way.” | | |
|  | I1* | | “The differences between fellows and residents are unclear.” | | |
|  | I2* | | “There are few objective criteria what a [rotation]resident should be able to do, and what not” | | |
|  | I4 | | “I often know little about the expectations that the residents have of their ICU-time”. | | |
|  | I9 | | “Experienced trainees, mostly fellows often work relatively independent, so that possible deficits or opportunities for improvement are little observed, and they may not be able to achieve the maximum possible, progress based on their level of knowledge / competence. | | |
|  | I11 | | “Heterogeneity in experience and primary discipline is sometimes challenging to define what can be expected (applies to residents, not to fellows)” | | |
|  | I13 | | “Heterogeneous trainees (internal medicine, surgery, anaesthesia, IPS fellows) with correspondingly heterogeneous previous knowledge, so that in everyday life, with an often high workload, individual needs cannot be adequately addressed”. | | |
|  | | | **2Sub4: the wish for a digital assessment tool (can make assessing easy and fun)** | | |
|  | R7 | | “I find a digital solution a la prEPAred App helpful and desirable. By this application, it is very easy to collect feedback. E.g., a compulsory assessment after each shift 🡪 your profile is always up to date, which may be useful for intensivists as well.” | | |
|  | F7 | | “We need to gain routine in practical activities (punctures, CVC insertions, etc.). Yet, case numbers are low” | | |
|  | F13 | | “There is also a need for a feedback tool (e.g. the "resident evaluation tool (RET)"), especially because of the constantly changing intensivists.” | | |
|  | I1* | | “We should find the right tool (for assessment).” | | |
|  | I14 | | “No standardised continuous documentation of the assessment including recommendations (e.g. More interventions) or emphasis on the positive aspects that can be further promoted.” [we need the] “prEPAred App (online assessment)” | | |
|  | | | **2Sub5: A dashboard can visualise learning progression, level of competence, strengths, weaknesses, goals / recommendations, and be able to create a solid based for summative assessment.** | | |
|  | F3 | | “Perhaps there should be an internal logbook of which fellow is allowed to do what under indirect or without supervision”. | | |
|  | F12 | | “Before a patient admission or an intervention, the level of knowledge of the counterpart is not discussed. Therefore, knowledge cannot be classified accordingly, and new learning objectives cannot be defined.” | | |
|  | F13 | | “A procedural catalogue, that should be fulfilled within the first 3 months. This can “approve” the trainee. E.g., a specified number of supervised procedures, after which an intensivist confirms that the trainee performs the intervention safely and according to guidelines and may work independently in the future. | | |
|  | I7 | | “Exchange between intensivists is important (regarding trainee-assessment).” | | |
|  | I13 | | “Due to the number of rotation residents, there is no overview of what the individual residents can and what they should and want to learn specifically.” | | |
|  | I14 | | “Lack of knowledge of the mentor’s interim evaluations and thus the goals and possible points of criticism” | | |
|  | | | **2Sub6: Competence committees should conduct the summative assessment** | | |
|  | I7 | | “Exchange between different supervisors is important (more regular meetings?) [to optimise assessment] | | |
|  | I8 | | [we need a] “platform for sharing the [summative] assessment” | | |
|  | I14 | | “No exclusive mentoring, but assessment of trainees in the team or at least exchange in the team before [summative] evaluations” | | |
|  | I15 | | “Perhaps in some cases (e.g., fellows), an oral exchange among the intensivists about the performance would also help. In discussions, I can formulate my impression even better than in written form – although this kind of discussion is very time-consuming.” | | |
| **Theme No.3: Insufficient coaching and -feedback lead to inadequate assessment** | | | | | |
| Definition: both insufficient coaching and insufficient feedback result in inadequate assessment | | | | | |
|  | R9 | | “Know the individual training status and based on this status, give appropriate and useful feedback.” | | |
|  | F3 | | “We need regular feedback (also negative-)” | | |
|  | F8 | | “I see the challenge in ensuring that the feedback is representative. Many trainees do not work frequently with all intensivist and so each intensivist has only seen a sample. For the trainee to get constructive, representative feedback, ALL intensivists need to give an assessment, not just 2-3” | | |
|  | F8 | | “I also see the challenge in ensuring that the feedback is constructive. A “you’re doing well” doesn’t help much; concrete situations need to be addressed.” | | |
|  | F11 | | “Feedback from nurses regarding visit structure/procedure is probably much more meaningful” | | |
|  | F11 | | “Interval-feedback missing, therefore the possibility to respond to criticism is lacking” | | |
|  | I3 | | “The assessments that are requested for the end [summative] assessment are perhaps somewhat one-dimensional”. | | |
|  | I8 | | “Mentoring functions stepmotherly. You must actively ask colleagues for a good overview. Scheduled meetings with mentee are frequently planned either too early or too late” | | |
|  | I12 | | “Overall, the mentoring [tutoring] system does not work well enough. As a mentor [tutor], I ask every two-three month how my mentees are doing and where I can support them. Sometimes I receive an answer, sometimes not. Overall, I still feel that I can only perform my mentor function to a certain extent, as I know too little about the performance of my mentees.” | | |
|  | | | **3Sub1: Hierarchy and conflicting roles (competing interests)** | | |
|  | R6 | | “Another difficulty is the prioritisation of Fellows. Of course, I understand that fellows need certain interventions, but I don't find the approach quite fair that the rotation residents are good for the paperwork, but not when it comes to interventions. That's a bit of an exaggeration, but it's the attitude of some fellows. What might help is if the interventions were carried out consistently by the trainees. I have repeatedly observed that the attending intensivist was "just up for" the intervention or hadn't done it for a "long time". | | |
|  | R7 | | “Relationship between fellows and residents leads to equal duties, yet unequal rights. I do not always find this a priori fair, especially when inexperienced fellows are involved. We [residents] have no chance for certain interventions (especially tracheostoma, also thoracic drainage and pulmonary catheters) despite the goal [for specialisation] of intensive care medicine and possibly a more advanced level of training than fellows. I have no problem with being in the second row, yet I find it a pity not to be allowed to stand in line at all (or only secretly).” | | |
|  |  | | **3Sub2: different personality traits may lead to misinterpretation** | | |
|  | F12 | | “Respond to the needs of trainees. Some stalk forward carelessly and full of energy. Others need more protection and support - this may also be asked for if not clearly felt.” | | |
|  | I2* | | “There is little acceptance by the trainee of a critical assessment, it is quickly judges as an attack”. | | |
|  | I14 | | “Subjective component: Personality of the trainee (e.g., if rather introverted) complicates assessment, trainees also contribute more or less depending on the personality of the intensivist” | | |
|  | | | **3Sub4: Coaches should be experienced and trained by faculty development, in which the art of giving and receiving feedback is taught**. | | |
|  | | I8 | “Intensivists are not schooled in leadership” | | |
|  | | I12 | “I try to give feedback (both positive and potential for improvement) to all trainees as often as possible and promptly after a shift. However, it takes time, the right environment and circumstances and requires skilful handling of people.” | | |
|  | | I12 | “I would welcome feedback for us mentors [tutors] from time to time on how to constructively challenge and encourage my trainees. (e.g. your email on how to give feedback was great, more of that please).” | | |
|  | | I15 | “In my new role (as intensivist), I am sometimes still very focused on "myself" and the new tasks. So, it is sometimes difficult for me to observe and judge trainees during their job.” | | |
|  | | | **3Sub5: define short term goals or competencies to achieve during short collaboration** | | |
|  | R4 | | “Assessment not possible in the absence of continuous care [from intensivist] and often absence [of the intensivist] for ward-rounds or invasive procedures” | | |
|  | F3 | | “I would be grateful that it will be announced, when knowledge is queried” | | |
|  | I8 | | “Scheduled talks [with trainee] are either too early or too late.” | | |
|  | I12 | | “Regular structured (knowledge, skills, behaviour in everyday life and intensivist suitability) assessment of the trainee by intensivist would help me as a tutor to give feedback to my assigned trainee.” | | |
|  | I12 | | “I try to give feedback (both positive and potential for improvement) to all trainees as often as possible and promptly after a shift.” | | |
|  | | | **3Sub6: A coach / mentor should inform themselves actively regarding the progression of their mentees** | | |
|  | | F1 | “If the mentor keeps actively checking in with the other intensivists about his or her mentees, at least the mentor should get a relatively good picture of the trainee assigned to him or her”. | | |
|  | | F8 | “If a questionnaire is sent to all intensivist before a trainee leaves the ICU and they comment in writing on the trainee: the person conducting the last assessment-interview MUST read through all the assessments first before giving them to the trainee to read. There may be personal and non-constructive evaluations which can then influence the trainee very strongly. Perhaps such evaluations do not need to be given to the trainees to read.” | | |
|  | | F9 | “A very big concern for me would be to receive regular feedback from mentors / intensivists on what needs to be improved. Proposal: At [another hospital], there is also a mentoring programme. One has regular discussions there with those intensivists with whom one works the most, regularly (= approx. 6-8x / year) fills out an assessment-sheet. The final evaluation and work certificate is based on these assessment-sheets. In this way, one has the possibility to react early and adequately to grievances and is not surprised with bad evaluations at the end. | | |
|  | | I8 | “All supervisors should feedback the mentor” [tutor, regarding strengths, weaknesses, and goals of their trainee] | | |
|  | | I12 | “Regular structured (knowledge, skills, behaviour in everyday life and intensivist suitability) assessment of the trainee by intensivist would help me as a tutor to give feedback to my assigned trainee.” | | |
|  | | I14 | “Lack of knowledge of the mentor's interim evaluations and thus the goals and possible points of criticism” | | |

This table provides an overview of relevant quotes that belong to the different themes and subthemes. Words within brackets [] are mentioned to clarify the statements to the reader.

*Note: Intensivist 1 is Author JBS and Intensivist 2 is author JCS

Abbreviations: F, fellow. FTE fulltime equivalent. I, intensivist. ICU, intensive care unit. R, resident. Sub, subtheme. WPBA, workplace-based assessment
